# Reduced Expression of Urokinase Plasminogen Activator in Brown Adipose Tissue of Obese Mouse Models

**DOI:** 10.3390/ijms22073407

**Published:** 2021-03-26

**Authors:** Chung-Ze Wu, Li-Chien Chang, Chao-Wen Cheng, Te-Chao Fang, Yuh-Feng Lin, Dee Pei, Jin-Shuen Chen

**Affiliations:** 1 Division of Endocrinology and Metabolism, Department of Internal Medicine, School of Medicine, College of Medicine, Taipei Medical University, Taipei 11031, Taiwan; chungze@yahoo.com.tw; 2 Division of Endocrinology and Metabolism, Department of Internal Medicine, Shuang Ho Hospital, Taipei Medical University, New Taipei City 23561, Taiwan; 3 School of Pharmacy, National Defense Medical Center, Taipei 11490, Taiwan; lichien@ndmctsgh.edu.tw; 4 Graduate Institute of Clinical Medicine, College of Medicine, Taipei Medical University, Taipei 11031, Taiwan; ccheng@tmu.edu.tw (C.-W.C.); linyf@shh.org.tw (Y.-F.L.); 5 TMU Research Center of Urology and Kidney, Taipei Medical University, Taipei 11031, Taiwan; fangtc@tmu.edu.tw; 6 Division of Nephrology, Department of Internal Medicine, School of Medicine, College of Medicine, Taipei Medical University, Taipei 11031, Taiwan; 7 Division of Nephrology, Department of Internal Medicine, Taipei Medical University Hospital, Taipei 11031, Taiwan; 8 Deputy Superintendent, Shuang Ho Hospital, Taipei Medical University, New Taipei City 23561, Taiwan; 9 School of Medicine, College of Medicine, Fu Jen Catholic University, New Taipei City 24205, Taiwan; peidee@gmail.com; 10 Division of Endocrinology and Metabolism, Department of Internal Medicine, Fu Jen Catholic University Hospital, New Taipei City 24352, Taiwan; 11 Department of Medical Education and Research, Kaohsiung Veterans General Hospital, No 386, Dazhong 1st Rd., Zuoying Dist., Kaohsiung City 81362, Taiwan; 12 Division of Nephrology, Department of Internal Medicine, Tri-Service General Hospital, National Defense Medical Center, Taipei 11490, Taiwan

**Keywords:** urokinase plasminogen activator, brown adipose tissue, obesity

## Abstract

In recent decades, the obesity epidemic has resulted in morbidity and mortality rates increasing globally. In this study, using obese mouse models, we investigated the relationship among urokinase plasminogen activator (uPA), metabolic disorders, glomerular filtration rate, and adipose tissues. Two groups, each comprised of C57BL/6J and BALB/c male mice, were fed a chow diet (CD) and a high fat diet (HFD), respectively. Within the two HFD groups, half of each group were euthanized at 8 weeks (W8) or 16 weeks (W16). Blood, urine and adipose tissues were collected and harvested for evaluation of the effects of obesity. In both mouse models, triglyceride with insulin resistance and body weight increased with duration when fed a HFD in comparison to those in the groups on a CD. In both C57BL/6J and BALB/c HFD mice, levels of serum uPA initially increased significantly in the W8 group, and then the increment decreased in the W16 group. The glomerular filtration rate declined in both HFD groups. The expression of uPA significantly decreased in brown adipose tissue (BAT), but not in white adipose tissue, when compared with that in the CD group. The results suggest a decline in the expression of uPA in BAT in obese m models as the serum uPA increases. There is possibly an association with BAT fibrosis and dysfunction, which may need further study.

## 1. Introduction

In recent decades, along with high-fat diets (HFD) and sedentary lifestyles, the prevalence of obesity has increased, resulting in a worldwide epidemic of type 2 diabetes mellitus, hypertension and cardiovascular events [1]. Obesity also contributes to non-alcoholic steatohepatitis progressing to liver cirrhosis and hepatoma [2], and leads to glomerular hyperfiltration, reduction of nephron mass, and glomerulopathy in the kidney [3]. Although the causality of obesity is complex, the excessive accumulation of fat mass is believed to be a crucial factor. Generally, there are two different types of adipose tissues—white adipose tissue (WAT) and brown adipose tissue (BAT) [4]. WAT is distributed in subcutaneous and peri-visceral regions and is mainly responsible for energy storage. BAT, containing abundant mitochondria in adipocytes, contributes to non-shivering thermogenesis, which can burn up to 20% of daily energy intake per 50 g of BAT. Recently, several studies have focused on BAT and its anti-obesity effect [5].

In the pathology of obesity-related complications, in the islets of patients with type 2 diabetes mellitus, amyloid deposition is a critical feature associated with loss of β cell mass [6]. Non-alcoholic steatohepatitis, in its aggressive necro-inflammatory form, may accumulate fibrosis, resulting in cirrhosis and end-stage liver disease [2]. Although most patients with obesity-related glomerulopathy have stable or slowly progressive proteinuria, up to one-third develop progressive renal failure and end-stage renal disease [7]. Recently, dysfunction of adipose tissue has been widely noted to be associated with hyperglycemia, dyslipidemia, and macrophage infiltration in peri-visceral fat [8,9,10]. Meanwhile, hypoxia and fibrosis of adipose tissue, abundant collagen, and extracellular matrix deposits contributing to inflammation and the infiltration of macrophages are regarded as important pathogenic mechanisms [11,12]. However, the exact pathophysiology of major organs with regard to adipose tissue fibrosis remains unclear.

Urokinase plasminogen activator (uPA), well known as a fibrinolytic protein, binds to its receptor (uPAR) and activates plasminogen, converting it to plasmin in the fibrinolytic process of thrombosis in the extracellular matrix. Apart from its functions in the fibrinolytic cascade, uPA is a pluripotent protease participating in activating the innate immune response, which regulates immune cell migration, recruitment, and lymphocytes proliferation [13,14]. Kawao et al. found uPA to play an important role in the activation of macrophage phagocytosis during liver repair [15]. In our previous study, we found uPAR to be related to various forms of kidney disease and its soluble form to be associated with the glomerular filtration rate and the amount of proteinuria present [16]. To date, the role of uPA in major organs during the development of obesity and adipose tissue dysfunction remains unknown.

It is essential to clarify the role of uPA in the causation of obesity-related complications. In the present study, we investigated uPA expression in WAT, BAT and major organs during the progression of obesity.

## 2. Results

### 2.1. The Different Presentations of Obesity and Metabolic Profiles in C57BL/6J and BALB/c Mice on HFD

In Table 1, we summarize the general characteristics of C57BL/6J and BALB/c mice in both groups. In the HFD group of C57BL/6J mice, body weight (BW) and total cholesterol (TC) increased over 8 weeks (W8). As time progressed, by 16 weeks (W16), levels of triglyceride (TG), and homeostasis model assessment–insulin resistance (HOMA-IR) were significantly elevated in the HFD group of C57BL/6J mice. The fractional excretion of sodium (FENa) was slightly decreased in the HFD group of C57BL/6J mice. Accordingly, the C57BL/6J HFD mice were prone to obesity, insulin resistance, glomerular hypofiltration, and dyslipidemia on a HFD. On the other hand, initially in BALB/c mice, metabolic profiles of the chow diet (CD) group and the HFD group did not differ significantly. However, by W16, BW, blood glucose (BG), TG, and HOMA-IR significantly increased, but FENa decreased in the HFD group of BALB/c mice. In addition, the presentation of renal function in BALB/c mice were assessed. The blood urea nitrogen (BUN) levels showed no significant difference in CD and HFD BALB/c mice (58.7 ± 14.1 ng/mL vs. 69.6 ± 7.7 ng/mL; *p* = 0.166). The proteinuria (urine protein/urine creatinine ratio) significantly increased in HFD BALB/c mice at W8 (1.98 ± 0.96; 3.93 ± 2.59; *p* = 0.038). However, the significant increase in proteinuria was not found by W16. BALB/c HFD mice were prone to obesity, insulin resistance, hyperglycemia, glomerular hypofiltraion and dyslipidemia. In our findings, the dysmetabolic phenotypes of obese mice induced by a HFD differed according to the strain.

### 2.2. Circulating uPA, Soluble uPAR (suPAR) and Plasminogen Activator Inhibitor-1 (PAI-1) Levels and Adiponectin in C57BL/6J and BALB/c Obese Mice

Table 1 also shows the serum uPA, suPAR, PAI-1 and adiponectin levels in C57BL/6J and BALB/c mice on a CD or a HFD. The uPA levels in the HFD group of both strains of mice were significantly increased at W8. However, at W16, the uPA levels of the CD and HFD groups in both strains of mice did not differ significantly. On the other hand, the suPAR levels were similar between the CD and HFD groups in both strains of mice at both W8 and W16. The PAI-1 levels in BALB/c mice were obviously higher than those in C57BL/6J mice. However, the PAI-1 levels significantly increased in BALB/c mice with HFD at both W8 and W16. Similar changes were not found in C57BL/6J mice. The adiponectin levels showed a significant decrease in the HFD W16 group in C57BL/6J mice and in the HFD W8 group in BALB/c mice in comparison to related CD groups.

### 2.3. uPA Expression on Subcutaneous White Adipose Tissue (sWAT), Visceral White Adipose Tissue (vWAT), BAT, Liver, Kidney, and Pancreas in C57BL/6J and BALB/c Obese Mice

**Figure 1:**
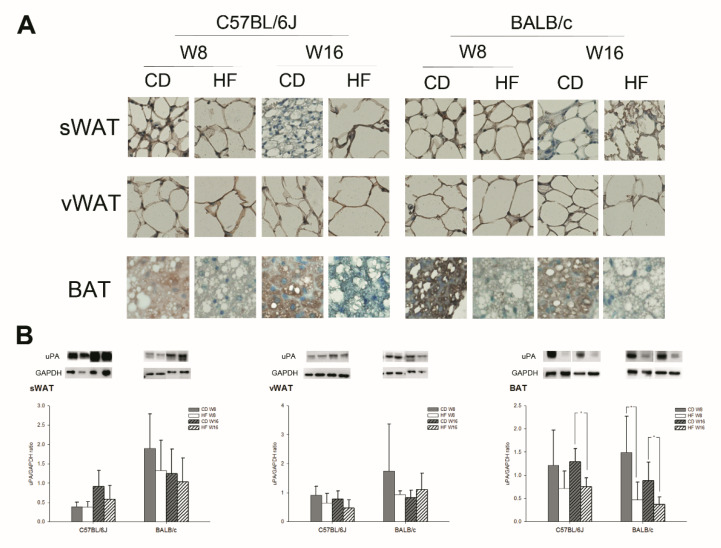
The (**A**) immunohistochemical stain and (**B**) Western blot for uPA expression on subcutaneous white adipose tissue (sWAT), visceral white adipose tissue (vWAT) and brown adipose tissue (BAT) in C57BL/6J and BALB/c mice (*n* = 5, in each group). The uPA expression in sWAT and vWAT of CD and HFD groups did not differ significantly, but there was a significant decline in the BAT in the HFD group. All figures of IHC stain: 400×.

**Figure 2:**
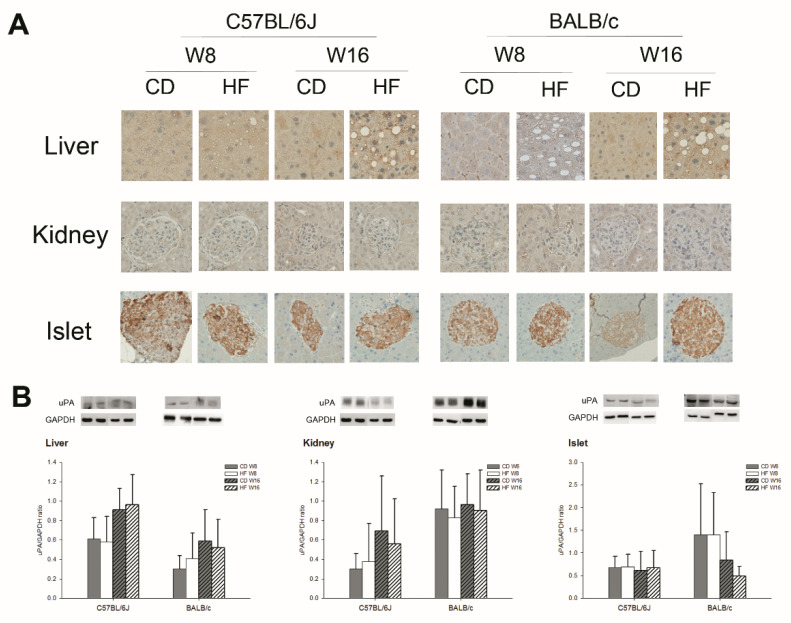
The (**A**) immunohistochemical stain and (**B**) Western blot for uPA expression on liver, kidney and islet tissue in C57BL/6J and BALB/c mice (*n* = 5, in each group). In the CD and HFD groups in both strains of mice, the uPA expression in the liver, kidney, and islet did not differ significantly. All figures of IHC stain: 400×.

Figure 1 and Figure 2 show the expression of uPA in various adipose tissues, the liver, the kidney, and the islets in C57BL/6J and BALB/c obese mice. In gross appearance, the size of fat droplets in sWAT, vWAT, BAT and the liver in the HFD group were larger than those in the CD groups in both strains of mice. In addition, the histology of the kidney showed no obvious change (Supplementary Materials Figure S1). The collagen IV expression of the liver mildly increased in HFD groups at W16 in both strains of mice (Figure S1). According to the findings of the immunohistochemistry (IHC) stain and Western blot (WB) for uPA, uPA expression in the liver, kidney, islet, sWAT and vWAT did not differ significantly between CD and HFD groups in either strain of mice. The uPA expression in the islet is mainly on β cells (Figure S2). In the kidney, the majority of uPA expression is on the tubular region. The uPA expression on the renal glomerular region is weak. However, the uPA expression in the kidney showed no prominent difference between CD and HFD groups in both strains of mice. Interestingly, we found a significant decline in uPA expression in BAT by IHC stain and WB early at W8, which persisted at W16 in the HFD groups in both strains of mice.

## 3. Discussion

In the present study, in different strains of HFD mice, we found different presentations of dysmetabolic profiles. Serum uPA levels initially increased and then declined with time in both C57BL/6J and BALB/c mice fed a HFD. In both C57BL/6J and BALB/c mice, the expression of uPA in BAT significantly declined after feeding a HFD. However, with a CD and a HFD, uPA expression was similar in the liver, kidney and WAT in both strains of mice. In HFD mice, uPAR expression in BAT and WAT showed no significant change.

C57BL/6J and BALB/c mice are commonly used for studies of mouse strains on immunoregulation in various disease models. As C57BL/6J mice preferentially develop the Th1 immune response and BALB/c mice, Th2-type cytokine polarization, they are regarded as prototypic Th1- and Th2-type mouse strains, respectively [17,18]. In addition to their distinct T-cell responses, macrophages from these two mouse strains exert different reactions in response to various stimuli [19]. Recent evidence indicates that the balance between the M1/M2 macrophages and the Th1/Th2 lymphocytes is of critical importance for the outcome of many diseases, including obesity-related metabolic disorders [20]. Jovicic et al. explored liver steatosis and immune cells in C57BL/6J and BALB/c mice fed a HFD for 24 weeks and found different immune–metabolic profiles between the two strains of mice [21]. C57BL/6J mice fed with a HFD were prone to obesity, hyperglycemia, increasing visceral adipose tissue, liver inflammation, and fibrosis. BALB/c mice fed with a HFD were susceptible to liver steatosis. Our findings do not contradict those of the empirical studies discussed above. BW significantly increased and FENa decreased in C57BL/6J on a HFD, which may imply that HFD-induced obesity, dyslipidemia and glomerular hypofiltration easily develop in Th1-prone mice. Contrastingly, in BALB/c mice fed a HFD, the increment in BG, TG and HOMA-IR significantly increased, and FENa decreased, which may imply that HFD-induced hyperglycemia, hypertriglycemia, insulin resistance and glomerular hypofiltration may be easily found on Th2-prone mice. In addition, adiponectin levels significantly decreased at W8 in HFD BALB/c mice, but not until W16 in HFD C57BL/6J mice. Adiponectin, one of the anti-inflammatory adipokines, decreases in inflammatory status. According to our results, it is suggested that HFD-related pro-inflammation may be found in the early phase of obesity in Th2-prone mice and in the late phase of obesity in Th1-prone mice.

The uPA is secreted from various cells and contributes to the degradation of the extracellular matrix and cellular remodeling and repair. Circulating uPA is predominantly excreted from hemopoietic cells and is responsible for the cascade activation of the fibrinolytic process and immune modulation [22]. The relationship between the uPA/uPAR system and atherosclerosis has been researched extensively. Some studies indicate high circulating uPA levels as possibly being involved in the migration of foamy cells or the stability of atheroma [23,24,25]. Our results indicate a significant increase in uPA in the early phase of obesity in both strains of mice. It was speculated that an increase in uPA may be a passive response to the accumulation of the extracellular matrix around endothelial cells and the modulation of fibrinogenesis and fibrinolysis. However, in our study, the increment in circulating uPA levels decreased in the late phase of obesity. It was presumed that some subsequent obesity-related inflammatory cytokines, such as PAI-1, might have inhibited the circulating uPA. Moreover, Zhou et al. investigated the change in circulating uPA in patients with chronic hepatitis B. The uPA levels increased in the acute phase but decreased in the late phase of hepatitis [26]. The findings are similar to the results of our HFD mice. They also highlighted the association between inflammation and uPA change. However, the real mechanism of uPA level change needs further study. Although some studies showed PAI-1 to be associated with adipocyte differentiation and regulating recruitment of inflammatory cells within adipose tissue [27], we found that serum PAI-1 levels of CD and HFD mice did not differ significantly in C57BL/6J mice. However, the PAI-1 levels significantly increased in HFD BLAB/c mice. In our previous studies of clinical investigation, PAI-1 was found to be positively related to the BMI percentile in boys and to body fat in girls [28]. The majority of studies explored PAI-1 expression in different tissues with an obese model. Morange et al. found that serum PAI-1 increased in obese mice fed a HFD for 17 weeks [29] which was similar to our BALB/c mice. Presumably, because the time frame over which we induced obesity in our C57BL/6J HFD mice was relatively short, the change in PAI-1 might not have had time to manifest in our C57BL/6J obese mice. In addition, PAI-1 is an inhibitor of plasminogen activator. PAI-1 levels non-significantly increased at W16 in HFD C57BL/6J mice and significantly increased at W16 in HFD BALB/c mice, which may also explain the decreasing uPA levels in both strains of mice at W16.

During the course of obesity development, WAT mass and the cellular size of adipocytes expand rapidly in the body. The rapid growth impedes the prompt delivery of sufficient oxygen to WAT from circulation, resulting in WAT dysfunction [30]. Consequently, several cytokines released from adipocytes induce systemic pro-inflammation and WAT fibrosis [9,12]. On the other hand, enlarged intracellular fat droplets and the reduced number of mitochondria in brown adipocytes have been noted during the process of obesity [31]. Obesity-related molecules involved in pro-inflammation and extracellular matrix turnover deteriorate BAT function [32]. Fibrotic BAT may exacerbate the development of obesity. We found decreasing expression of uPA in the BAT of obese mice in both strains. It was assumed that the decline in uPA expression in the BAT may contribute to adipocyte fibrosis and dysfunction, in turn leading to obesity-related complications. Spencer et al. also found increased collagen V expression in adipose tissue in obese subjects and presumed the extracellular matrix to be associated with insulin resistance [33]. However, an insufficiency of uPA in BAT would impair the necessary degradation of the extracellular matrix, resulting in the compromised migration and remodeling of brown adipocytes. Although we did not detect histologic fibrosis of BAT in our mice, other studies had similar findings. Alcalá et al. investigated mice fed with a HFD for 20 weeks, during which they developed obesity and mild hyperglycemia [34]. The pathology of BAT in HFD mice showed cellular hypertrophy and no obvious fibrotic change. However, they found inflammation, oxidative stress, and some anti-oxidative enzyme activity reflectively increased in BAT in HFD mice. In addition, Trayhurn et al. found an increase in some markers related to fibrosis during deoxygenation following BAT expansion in obesity [30]. Consequently, uPA may have responded before the formation of histologic fibrosis in the BAT. However, further research into the cause of uPA decline in BAT on a HFD is necessary.

There are some limitations to our study. First, several factors may have influenced the activation of BAT, including cold temperatures and adrenergic stimulation [35]. Our mice were caged at room temperature during the whole period of the experiment so that we did not observe changes in uPA expression in the BAT in cold temperatures. Second, the real mechanism of decreasing uPA expression on BAT, not WAT, on a HFD remains unknown. We presume that lipotoxicity in brown adipocytes may be one possible factor. Further study of the cellular model is needed to explore the exact pathway. Third, our numbers of mice were only five per group. The statistic power of serum biochemistries in each group of mice may not be enough. However, the trends of metabolic parameters between CD and HFD groups in different strands of mice may be informative. Fourth, we did not have the data of baseline biochemistries in each group of mice for assessing the change in each variable. However, there were two points of time in each group for evaluating the difference during the treating interval. In addition, we did not explore the change in various organs after obesity. The hypertrophy of an organ after obesity may be associated with uPA expression. However, our research is the first to explore the relationships between uPA changes and adipose tissue in obesity. We used strains of mice with different immune-prone characteristics to arrive at our circulating and histologic findings. Whether changes in uPA in the BAT induced BAT dysfunction in obesity or the two phenomena develop simultaneously is a worthy subject for advanced studies in the future.

## 4. Materials and Methods

### 4.1. Induced Obese Mouse Model

The male wild-type C57BL/6J and BALB/c mice, obtained from the Laboratory Animal Center (National Taiwan University College of Medicine, Taipei, Taiwan), Taiwan, were housed in laboratory cages and, from the age of 5 weeks, fed a normal CD in the CD group and a HFD (40% fat) in the HFD group, respectively. We measured BW and BG weekly. These mice were euthanized at W8 or W16 after feeding with a CD or HFD. Blood samples were collected before euthanasia. The sWAT from inguinal WAT, and vWAT from the WAT of the epididymis were harvested. The BAT from the posterior neck region, liver, kidney, and pancreatic tissues were harvested from the mice after euthanasia. The Institutional Animal Care and Use Committee at National Defense Medical Center, Taipei, Taiwan approved the experimental animal protocol (Approval No: IACUC-13-199).

### 4.2. Measurement of uPA, suPAR, PAI-1 and Metabolic Biochemistry

After collecting blood, we separated plasma and serum by centrifugation and stored them at −80 °C prior to analysis. The uPA was measured by using the Mouse uPA Total Antigen Assay enzyme-linked immunosorbent assay (ELISA) kit (Molecular Innovations, Novi, MI, USA), with the intra- and inter-assay coefficients of variation being 6.18% and 7.47%, respectively. The soluble uPAR were measured by using the Mouse uPAR DuoSet ELISA kit (DY531, R&D system, Minneapolis, MN, USA). The mean coefficient of variation in these assays was 5%. Murine PAI-1 was measured by using the PAI-1 Total Mouse ELISA kit (ab157529, Abcam, Cambridge, MA, USA) with the intra- and inter-assay coefficients of variation being 7.9% and 12.9%, respectively. Total cholesterol, TG, serum and urine sodium, and creatinine were analyzed by spectrophotometry (Fuji Dri-Chem 3000, Fuji Film, Kanagawa, Japan). The FENa was applied to evaluate renal filtration and calculated by the ratio of urine and plasma sodium and creatinine. Serum insulin was measured using the Mouse Insulin ELISA Kit (Mercodia AB, Uppsala, Sweden) with the intra- and inter-assay coefficients of variation being 3.4% and 3.6%, respectively. All samples were assayed in duplicate. HOMA-IR was calculated to assess insulin resistance [36].

### 4.3. IHC Stain and WB of sWAT, vWAT, BAT, Liver, Kidney, and Islet

The sWAT, vWAT, BAT, liver, kidney and pancreatic tissues were fixed in 10% formaldehyde fixative solution and embedded tPBShem in paraffin. The sections of formalin-fixed tissue were immersed in xylene for 5 min three times and incubated with phosphate-buffered saline (PBS) and 1% bovine serum albumin (BSA) at room temperature (RT) for 30 min for blocking. After removing paraffin and rehydrating, the slices were incubated with 1:400 dilution of the primary antibody (anti-uPA antibody (ab28230, Abcam, Cambridge, MA, USA)) in PBS at 4 °C overnight. Subsequently, the slices were incubated with 1:50 dilution of the secondary antibody (biotinylated anti-rabbit antibody (Vector Laboratories, BA-1300, CA, USA)) for 40 min and washed with Tris-buffered saline containing 0.05% Tween 20 (TBST; pH 7.4). We then treated the sections with VECTASTAIN ABC (Vector Laboratories, CA, USA) working solution for 30 min. The peroxidase activity was visualized with 3,3′-diaminobenzidine (DAB) using a DAB substrate kit for peroxidase (BD Pharmingen™). The slices were observed with an optical photomicroscope.

For WB, equal amounts of protein (30 μg) from each tissue of various whole organs were separated after homogenization by 8% SDS-PAGE gel, which was electro-blotted onto a nitrocellulose membrane and incubated for 1 h in blocking buffer (TBST, 2% bovine serum albumin). It was then washed three times in TBST and incubated with 1:2000 dilutions of anti-uPA antibody (ab28230, Abcam, Cambridge, MA, USA) or 1:10000 dilutions of anti-glyceraldehyde-3-phosphate dehydrogenase (GAPDH) antibody (ab181602, Abcam, Cambridge, MA, USA), respectively, in TBST at 4 °C overnight. The membranes were washed blots and incubated in horseradish peroxidase-conjugated goat-anti-rabbit-IgG-HRP antibody (Cat#3053-S-Ex, EPITOMICS, CA, USA) for 1 h at room temperature. After washing the membranes, we detected and incubated the membrane-bound antibody with a Western blot detection system and captured it on X-ray film.

### 4.4. Statistical Analysis

The PASW statistics version 18.0 package for Windows (IBM SPSS Statistics) was used for data analysis. The continuous variables were expressed as mean ± SD. A nonparametric Mann–Whitney U test was used for comparison of the two groups. All statistical data are expressed as two-sided, and *p* values < 0.05 considered to be statistically significant.

## 5. Conclusions

In summary, HFD-induced obesity presents different dysmetabolic profiles in different immune responses. Serum uPA increased in the early phase of obesity in our model. Decreasing uPA expression in the BAT may contribute to BAT dysfunction in obesity.

## Figures and Tables

**Table 1:** Relationship between glomerular hyperfiltration and metabolic status in C57BL/6J and BALB/c mice fed a chow diet (CD) or high fat diet (HFD) at 8 weeks (W8) and 16 weeks (W16). (*n* = 5, in each group).

	C57BL/6J				BALB/c			
	W8		W16		W8		W16	
	CD	HFD	CD	HFD	CD	HFD	CD	HFD
Body weight (g)	25.80 ± 0.94	29.46 ± 1.32 ***	27.04 ± 2.14	39.1 ± 1.86 ***	29.26 ± 2.05	30.22 ± 1.23	30.04 ± 3.30	33.95 ± 2.73 *
BG (mmol/L)	6.97 ± 1.22	9.24 ± 3.21	7.43 ± 0.90	9.50 ± 3.96	5.08 ± 1.00	6.36 ± 2.23	5.40 ± 1.25	6.96 ± 1.73 *
TC (mmol/L)	2.60 ± 0.03	3.12 ± 0.35 *	2.59 ± 0.01	3.68 ± 0.58 *	4.94 ± 0.44	4.92 ± 0.81	3.42 ± 0.06	4.24 ± 1.36
TG (mmol/L)	1.11 ± 0.26	1.19 ± 0.09	1.07 ± 0.19	1.34 ± 0.13 *	1.06 ± 0.09	1.07 ± 0.14	0.85 ± 0.11	0.71 ± 0.08 *
HOMA-IR	2.58 ± 1.00	5.42 ± 2.88	2.96 ± 1.03	8.05 ± 1.61 ***	1.80 ± 1.06	5.16 ± 3.75	1.76 ± 1.11	9.21 ± 5.92 *
HOMA-ß	26.42 ± 3.09	23.27 ± 6.06	41.30 ± 17.45	34.71 ± 18.95	258.4 ± 151.1	618.4 ± 502.5	366.8 ± 458.9	122.4 ± 64.8
FENa	1.00 ± 0.76	0.69 ± 0.48	1.08 ± 0.70	0.33 ± 0.24 **	0.81 ± 0.78	0.84 ± 0.83	1.14 ± 0.54	0.53 ± 0.37 *
uPA (μg/mL)	0.87 ± 0.35	2.56 ± 0.65 *	1.24 ± 0.55	1.92 ± 0.19	1.88 ± 0.48	3.34 ± 0.84 *	1.31 ± 0.37	2.52 ± 0.95
suPAR (ng/mL)	3.26 ± 0.19	3.43 ± 0.36	2.56 ± 0.74	2.27 ± 0.53	1.67 ± 0.60	2.22 ± 0.66	1.64 ± 0.56	3.09 ± 1.23
PAI-1 (ng/mL)	4.95 ± 0.77	4.06 ± 0.45	3.86 ± 0.58	7.76 ± 1.01	28.80 ± 2.03	36.22 ± 4.39 *	22.54 ± 2.12	56.16 ± 4.60 ***
Adiponectin	7.92 ± 0.43	9.06 ± 0.68	8.86 ± 0.45	6.98 ± 0.45 *	9.12 ± 0.38	6.96 ± 0.13 **	8.87 ± 0.45	8.31 ± 0.19

Data shown as mean ± SD; BG: blood glucose, TC: Total cholesterol, TG: Triglyceride, HOMA-IR: homeostatic model assessment–insulin resistance, HOMA-ß: homeostatic model assessment-ß, FENa: functional excretion of sodium; * *p* < 0.05, ** *p* < 0.01, *** *p* < 0.001, compared with the CD group.

## Supplementary Materials

The following are available online at https://www.mdpi.com/article/10.3390/ijms22073407/s1.
