# Supplementary material for: Reduced Expression of Urokinase Plasminogen Activator in Brown Adipose Tissue of Obese Mouse Models
Source: Int J Mol Sci. 2021 Mar 26;22(7):3407. doi: 10.3390/ijms22073407 (PMC8037769; doi:10.3390/ijms22073407)
Supplement: Supplementary file 1 [file ijms-22-03407-s001.pdf]

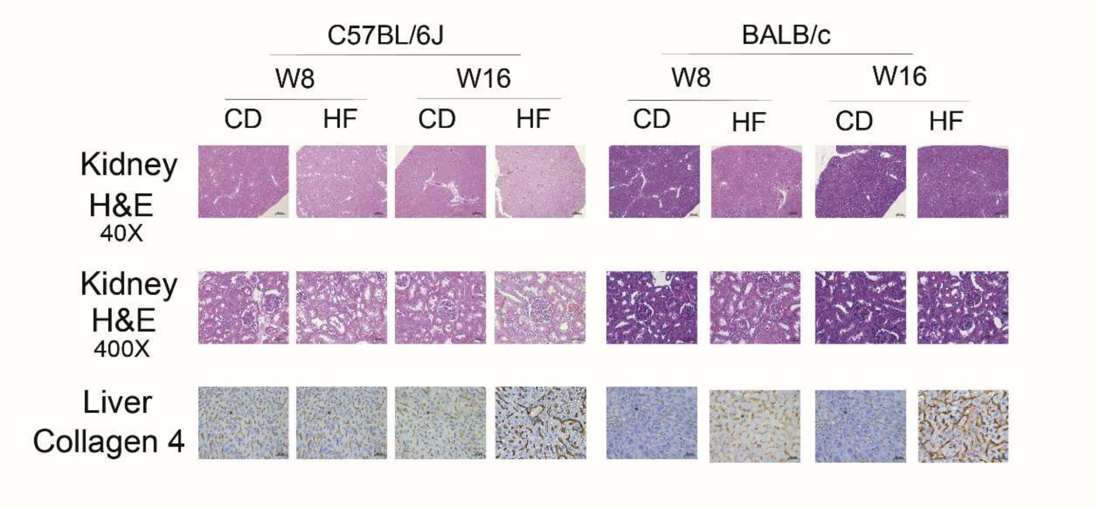

Figure S1 The H&E stain on kidney and IHC stain of collagen 4 in liver in C57BL/6J and BALB/c mice (n=5, in each group). In the gross histology of kidney showed no obvious change between CD and HFD groups in two strains of mice. The collagen 4 expression of liver prominently increased after HFD at W16 in both strains of mice.

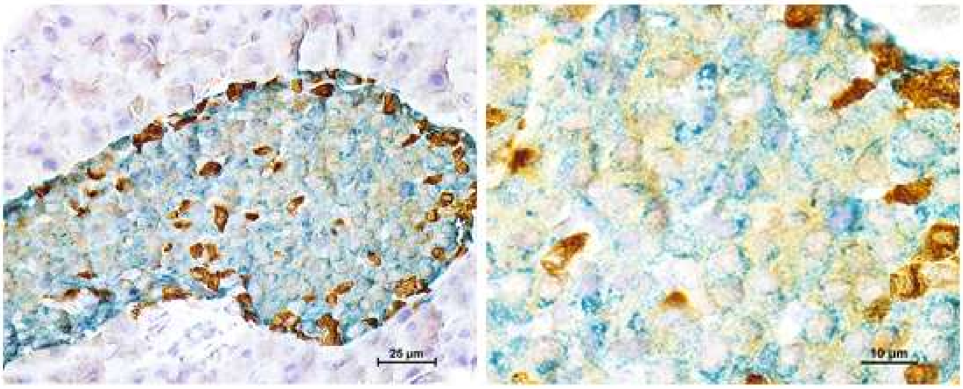

Figure S2 IHC triple stains of islet. (uPA: brown, Glucagon: pink, Insulin: green). The uPA expression is overlapped on insulin. Majority of uPA expression is on β cells.
